# Triage Assessment of Lateral Ankle Sprain Surgical Risk (TALAR Score): Using Early Red Flags to Predict the Failure of Conservative Management

**DOI:** 10.3390/jfmk11020223

**Published:** 2026-05-31

**Authors:** Raffaele Vitiello, Antonio Bove, Guglielmo Miele, Andrea De Fazio, Luca Magrini, Marianna Citro, Matteo Turchetta, Fabrizio Forconi

**Affiliations:** 1 Department of Orthopedics and Geriatric Sciences, Università Cattolica del Sacro Cuore, Largo Francesco Vito, 8, 00168 Rome, Italy; raffaele.vitiello@guest.policlinicogemelli.it (R.V.);; 2 Department of Aging, Orthopaedic and Rheumatological Sciences, Fondazione Policlinico Universitario Agostino Gemelli IRCCS, Largo A. Gemelli, 8, 00168 Rome, Italy; 3 Top Physio Clinics, 00136 Rome, Italy; 4 Villa Stuart Sport Clinic, FIFA Medical Center of Excellence, Via Trionfale 5952, 00135 Rome, Italy

**Keywords:** ankle sprain, chronic ankle instability, TALAR score, sports injuries

## Abstract

**Background:** Functional testing after an ankle sprain may help identify patients who later develop mechanical instability and require surgery. This study aimed to identify early clinical and functional predictors of surgical stabilization for chronic ankle instability (CAI) after acute sprains and to develop a simple composite predictive score (TALAR). **Methods:** This prospective observational study included 197 patients with acute lateral ankle sprains. Comprehensive clinical and functional assessments, including range of motion (ROM), strength, and pain, were performed two weeks post-injury. The primary outcome was subsequent surgical management for instability within a 24-month follow-up period. **Results:** Eight patients (4%) ultimately underwent surgical stabilization. Univariable analysis identified three significant predictors of surgical outcome: eversion mobility ≥ 20°, plantar flexor strength ≤ 17 kg, and the presence of pain during dorsiflexion (VAS > 0). These variables were integrated into the 0–3 TALAR (Triage Assessment of Lateral Ankle sprain Surgical Risk) score, which demonstrated promising exploratory discrimination with an AUC of 0.889 (95% CI: 0.799–0.954). An optimal cut-off of ≥2 yielded a sensitivity of 0.875 and a specificity of 0.822. While the baseline surgical risk was 4%, patients with a TALAR score ≥2 had a 17.5% conversion rate to surgery, representing a significantly higher risk (OR: 32.24; *p* < 0.001). **Conclusion:** The TALAR score represents a promising exploratory tool for early risk stratification after an acute ankle sprain. As an exploratory study, it highlights that early functional red flags, though formal internal and external validation, along with robust calibration on longer follow-up cohorts, are required before clinical implementation.

## 1. Introduction

Lateral ankle sprains (LAS) are amongst the most common injuries affecting the musculoskeletal system. In the general population, the estimated prevalence ranges from two to seven acute ankle sprains/1000 person-years [1]. Physically active people present an even higher incidence of ankle sprains, which represents about 15% of all sports-related traumas [2]. The true incidence of ankle sprains may be up to 5.5 times higher than emergency department estimates due to underreporting [3].

The typical injury mechanism of lateral ankle sprains involves a rapid inversion of the foot, often combined with plantarflexion, occurring shortly after ground contact during landing or cutting movements [4]. The anterior talofibular ligament (ATFL) is the most injured ligament, followed by the calcaneofibular ligament and the posterior talofibular ligament that are involved in more severe injuries [5].

Conservative management is widely regarded as the first-line treatment for LAS and it includes the POLICE (protected optimal loading ice compression elevation) often combined with multimodal physiotherapy to reduce pain and swelling promoting functional recovery [6]. In the acute phase following a LAS, current evidence supports early mobilization rather than temporary immobilization in a plaster cast for pain and swelling control [7]. After the reduction in pain and swelling, functional therapy is typically initiated using a semi-rigid ankle brace [8]. The role of kinesio taping is still debated with scientific evidence showing limited effectiveness in ankle sprain management [9].

Conservative management of LAS achieves complete recovery in about 36–85% of patients [10]. Primary surgical repair followed by functional rehabilitation is currently uncommon (0.5–4% of all LAS) and is generally reserved for severe injuries, such as complete rupture of the lateral ligament complex in elite athletes, combined lateral and medial ligament tears, unstable osteochondral fractures of the talus with free floaters in articulation, displaced avulsion fractures, open ligament injuries, or hemarthrosis associated with compartment syndrome [11].

Chronic ankle instability (CAI) may result from failure of conservative management of LAS involving an increased risk of recurrent sprains. CAI is a condition characterized by sensations of the ankle “giving way,” including persistent symptoms, such as pain, weakness, or limited range of motion, reduced function, and recurrent ankle sprains persisting for more than one year after the initial injury [12]. Mechanical laxity, neuromuscular deficits, and altered sensorimotor control may contribute to the development of CAI [1,13]. Some studies report that CAI may develop in up to 70% of cases following an ankle sprain, particularly in patients practicing sports [14].

Despite its high prevalence, CAI often remains under-recognized, partly due to non-specific symptoms and reduced activity without medical assessment [1,15]. CAI highly impacts patients’ quality of life since it is associated with persistent pain, functional deficits, reduced participation in sport and work, and an increased risk of post-traumatic ankle osteoarthritis [16,17]. Since ankle sprains generate a substantial healthcare financial burden, including significant direct and indirect costs, early identification of the appropriate treatment approach, conservative or surgical, following LAS may contribute to more efficient management of healthcare resources [18].

While the individual clinical and functional parameters associated with acute ankle sprains are well known to managing clinicians, their lack of integration into a structured therapeutic decision-making model makes it difficult to establish an early, objective indication for either conservative or surgical treatment of acute ankle sprains. Therefore, the aim of this exploratory study is to describe preliminary univariable associations between early clinical measures and subsequent surgical treatment, providing a first step toward an objective triage system.

## 2. Materials and Methods

### 2.1. Population

Between July 2022 and March 2024, 197 consecutive patients who underwent an acute ankle sprain (LAS) were enrolled after providing written informed consent. The clinical characteristics of the study population are detailed in Table 1. The study was conducted in accordance with the Declaration of Helsinki. Given the observational, non-interventional, and exploratory nature of the study, as well as the strict use of anonymized patient data, the protocol was reviewed and approved by the Internal Review Board of the Orthopaedic and Traumatology Institute of Università Cattolica del Sacro Cuore, Roma (Session of June 2022). In accordance with institutional guidelines for observational research on anonymized data, a formal external ethics committee protocol number was waived.

Inclusion criteria consisted of a documented history of acute ankle sprain, aged between 18 and 70 years, documented absence or history of previous sprains in the same ankle, standardized clinical and functional assessments, and a minimum follow-up period of 20 months. Exclusion criteria were a previous ankle surgery, neuromuscular disorders, incomplete baseline functional assessment.

### 2.2. Study Design

This prospective observational study followed a standardized clinical and functional assessment protocol aiming at identifying clinical and functional predictors associated with surgical indication in patients with LAS. Patients’ clinical assessment was performed two weeks after the ankle sprain injury. All participants underwent a comprehensive evaluation, including ankle range of motion (ROM), muscle strength testing, proprioceptive and balance assessment, surface electromyography (EMG) of the peroneal muscles and pain assessment during dynamic and functional tests. Patients were also asked to fill out the Foot and Ankle Ability Measure (FAAM) questionnaire to assess patient-reported health status. All patients were initially managed according to a standardized conservative protocol, including a bivalve brace for at least 3 weeks and progressive weight-bearing as tolerated. Physiotherapy was initiated 10–14 days after injury, focusing initially on edema control, pain reduction, and restoration of ROM, followed by progressive strengthening and proprioceptive re-education. Adherence to rehabilitation was encouraged and clinically reviewed during follow-up visits but was not quantitatively monitored.

### 2.3. Outcome and Candidate Predictors

The primary outcome was to describe univariable associations between clinical and functional predictors and subsequent surgical management for ankle instability. To minimize indication bias, the decision to proceed with surgical stabilization was not arbitrary but followed a standardized clinical protocol across the institution. Specifically, surgical management was indicated strictly for patients who demonstrated a failure of conservative treatment, defined as the persistence of mechanical instability and clinically significant symptoms (such as recurrent giving-way, persistent pain, and functional impairment) after a minimum of 3 months of targeted, multimodal physiotherapy and strict adherence to the rehabilitation protocol. Because the decision to proceed to surgery remains a clinical decision influenced by symptoms, functional demands, and patient expectations, this endpoint was interpreted as a pragmatic clinical outcome rather than a purely biological measure.

The clinical and functional parameters used as predictors are:

ROM: dorsiflexion, plantarflexion, inversion, and eversion measured with a goniometer.

Muscle strength: peroneal, dorsiflexor, and plantar flexor strength assessed using an isometric dynamometer. Results were recorded as absolute values (kg) and as percentage deficits relative to the contralateral limb ([(healthy − affected)/healthy] × 100).

Pain intensity: visual analog scale (VAS; 0–10) recorded during dynamic assessments and functional performance tests. Pain variables were dichotomized as presence or absence of pain.

Functional performance tests: Y-balance test, foot-lift test, side hop test (30 s repeated lateral jumps), and 6 m timed hop test.

Surface EMG: assessment of peroneal muscle activation to identify delayed recruitment or asymmetry.

FAAM questionnaire: to assess patient-reported health status.

All measurements were performed by the same team of orthopaedics with expertise to maximize inter-operator consistency. All patient’s data were registered on an Excel dataset.

### 2.4. Statistical Analysis and Score Development

All analyses were performed on the provided Excel dataset. Two groups of patients were initially identified: patients who subsequently underwent surgery for instability versus patients who did not have surgery. Continuous variables were summarized using median [interquartile range] and mean ± standard deviation, whereas categorical variables were presented as counts and percentages. Univariable group comparisons used Mann–Whitney U tests for continuous variables (two-sided); categorical variables were compared using Fisher’s exact test for 2 × 2 tables when expected counts were small and χ^2^ tests otherwise. Pain scores (VAS variables) were additionally transformed into dichotomous indicators (0 if VAS = 0; 1 if VAS > 0).

ROC analyses were performed for selected predictors by computing sensitivity and specificity across all observed thresholds; AUC was estimated via trapezoidal integration. The Youden index (J = sensitivity + specificity − 1) was used to identify optimal cut-offs. Univariable analyses were conducted to identify clinical, functional, and pain-related variables associated with subsequent surgical stabilization. Variables demonstrating statistically significant associations and clinical plausibility were selected as candidate predictors. Variables, such as surface EMG, Y-balance test, and FAAM questionnaire scores, were excluded from the final model because they did not exhibit statistically significant differences during univariable screening (all *p* > 0.05), and their exclusion preserves the score’s simplicity and rapid bedside applicability. Because of the limited number of surgical events, multivariable modeling was not performed. Instead, each candidate predictor was evaluated using ROC curve analysis, and optimal cut-off values were determined using the Youden index.

Because of the limited number of surgical events, multivariable modeling was not performed. Instead, each candidate predictor was evaluated using ROC curve analysis, and optimal cut-off values were determined using the Youden index.

A composite clinical prediction score—TALAR (Triage Assessment of Lateral Ankle Sprain Surgical Risk)—was developed as a discrete ordinal score based on dichotomized clinical variables. One point was assigned for each of the following findings: eversion mobility of the affected ankle ≥ 20°, plantar flexor strength of the affected ankle ≤ 17 kg, and presence of pain during dorsiflexion. The final score ranged from 0 to 3, with higher values indicating a greater number of early clinical red flags potentially associated with failure of conservative management. Dichotomization of continuous variables was chosen to improve clinical interpretability and facilitate bedside risk stratification, despite the acknowledged loss of statistical information. Therefore, the score was not intended as a definitive predictive model, but as a preliminary exploratory framework. The apparent discriminative ability of the TALAR score was assessed using ROC curve analysis. To account for optimism from deriving and testing the score in the same cohort, internal validation of the apparent AUC was performed using bootstrap resampling, and the mean optimism was subtracted from the apparent AUC to obtain an optimism-corrected AUC. The observed surgical conversion rate was also reported across TALAR score levels as a descriptive risk-gradient analysis. Given the exploratory design and small number of surgical events, these estimates were interpreted as hypothesis-generating rather than confirmatory.

## 3. Results

Between July 2022 and March 2024, 197 consecutive patients with LAS were included. Of these patients, 4% (eight patients) subsequently underwent surgical stabilization for ankle instability within 2 years from trauma, while 96% (189) were managed conservatively. The cohort had a mean age of 36.12 (±15.84) years and a mean BMI of 23.75 (±7.8). Demographic characteristics of the study cohort are summarized in Table 1.

Clinical and functional test results are summarized in Table 2.

Univariable screening of all available demographic, clinical, functional, and pain-related variables revealed no significant differences between the surgical and non-surgical groups for age, sex, BMI, ankle dorsiflexion, plantarflexion, inversion, or for most strength and pain measures (all *p* > 0.05) (Table 2).

Among range-of-motion variables, eversion mobility of the affected ankle was the only parameter significantly associated with subsequent surgical treatment. Median eversion was higher in the surgical group than in the non-surgical group, 20° [IQR 13.8–24] versus 10° [IQR 5–15], respectively, *p* = 0.0093. Regarding muscle performance, plantar flexor strength of the affected ankle was significantly lower in the surgical group, with a median value of 13 kg [IQR 10.8–15.5], compared with 20 kg [IQR 14–30] in the non-surgical group, *p* = 0.025.

Pain variables were also largely comparable between the two study groups; however, after dichotomization, VAS in dorsiflexion was significantly more frequent among surgical patients, being present in 62.5% compared with 16.9% of non-surgical patients (*p* = 0.006), as shown in Table 3.

**Figure 1:**
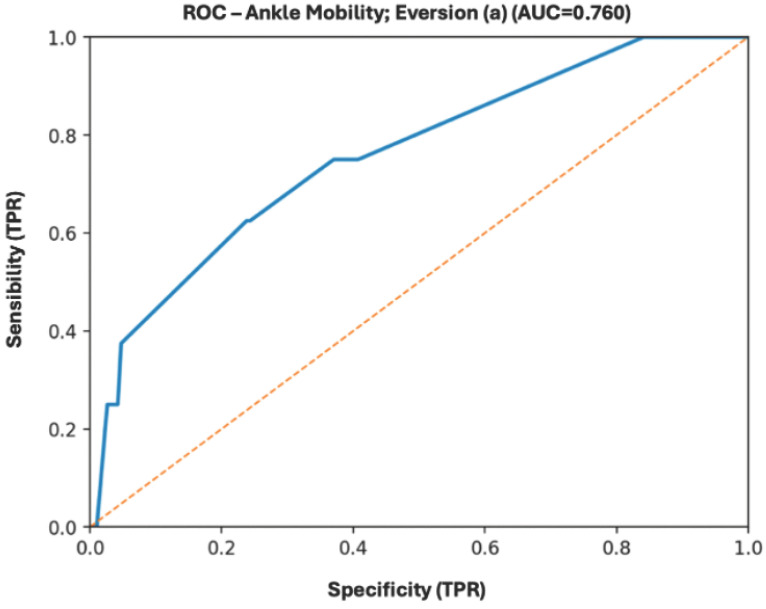
ROC analysis of Ankle eversion mobility.

**Figure 2:**
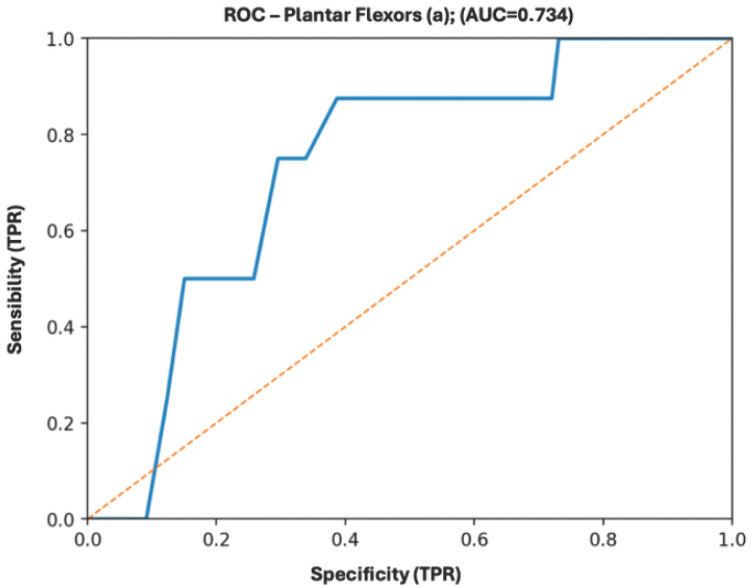
ROC analysis of plantar strength.

Plantar flexion strength, eversion mobility and pain during dorsiflexion were therefore selected as candidate predictors and further evaluated using ROC analysis. Eversion mobility demonstrated an AUC of 0.760 (95% CI 0.585–0.915), in Figure 1, with an optimal Youden cut-off of ≥20°; while plantar flexor strength showed an AUC of 0.734 (95% CI 0.571–0.858), in Figure 2, with an optimal Youden cut-off of ≤17 kg.

The TALAR composite score was developed; the score ranges from 0 to 3 and it is calculated by assigning one point for each of the following present at clinical assessment: eversion mobility of the affected ankle ≥20°, plantar flexor strength of the affected ankle ≤ 17 kg, and presence of pain during dorsiflexion.

**Figure 3:**
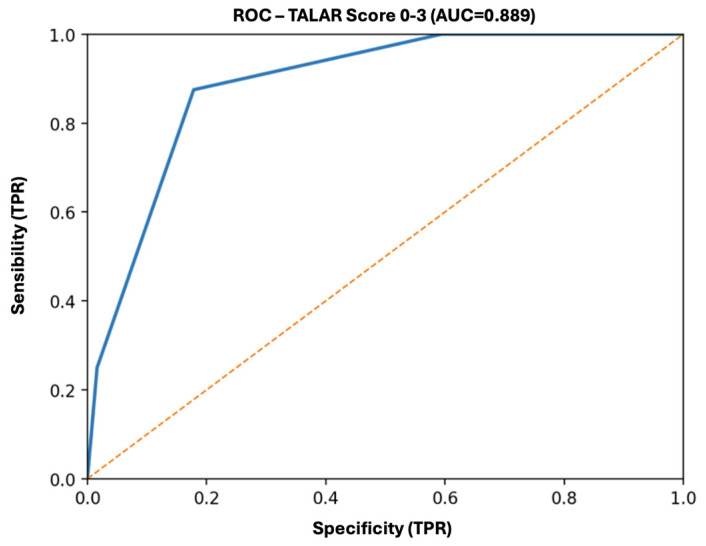
ROC analysis of TALAR score.

The TALAR score showed promising apparent discrimination for subsequent surgical stabilization, with an AUC of 0.889, 95% CI 0.799–0.954, Figure 3. Bootstrap internal validation showed a mean optimism of 0.033, resulting in an optimism-corrected AUC of 0.855. These findings suggest that the discriminative performance of the score remained promising after optimism correction, although the estimate should be interpreted cautiously because of the limited number of surgical events.

The optimal TALAR cut-off identified by the Youden index was ≥2. At this threshold, sensitivity was 0.875 and specificity was 0.822, Table 4. Patients with a TALAR score ≥ 2 had significantly higher odds of subsequent surgical stabilization compared with those with lower scores, OR 32.24, *p* = 7.1 × 10^−5^. While the baseline surgical risk in the overall cohort was 4.1%, patients with a TALAR score ≥ 2 showed a surgical conversion rate of 17.5%.

**Figure 4:**
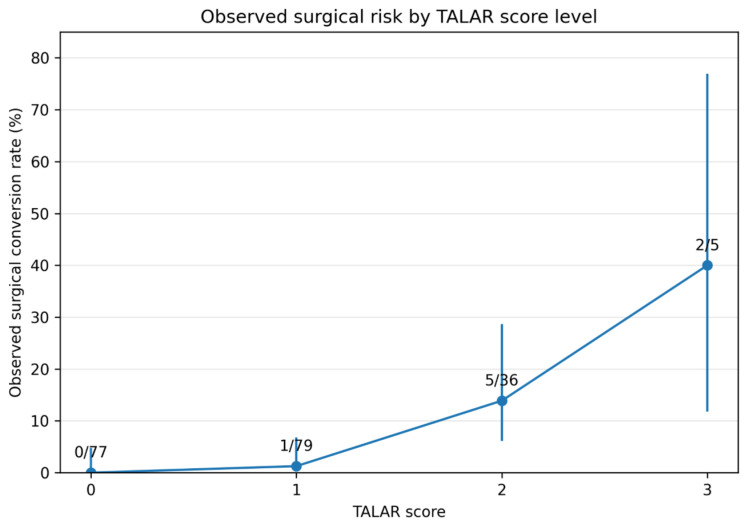
Observed surgical conversion rate according to TALAR score level.

The observed surgical conversion rate increased progressively across TALAR score levels. No surgical events were observed among patients with a score of 0. One of 79 patients with a score of 1 underwent surgery, corresponding to 1.3%. Five of 36 patients with a score of 2 underwent surgery, corresponding to 13.9%, and two of five patients with a score of 3 underwent surgery, corresponding to 40.0%, as in Figure 4. This gradient supports the exploratory value of the TALAR score as a preliminary risk-stratification framework, although estimates for higher score levels should be interpreted with caution because of the small number of events.

## 4. Discussion

Acute ankle sprain is a common injury with variable clinical evolution. A substantial number of patients continue to experience symptoms and functional instability despite adequate conservative care [10]. Early identification of patients at risk of requiring surgical treatment is crucial to optimize clinical decision-making, guide timely referral to specialized care, and potentially prevent prolonged symptoms and progression to chronic ankle instability.

The primary outcome of this study indicates that 4% of the total cohort ultimately required surgical intervention for instability. While most routine clinical variables failed to predict this outcome, eversion mobility, plantarflexor strength, and pain during dorsiflexion demonstrated significant prognostic value. These parameters, integrated into the TALAR score, achieved a promising apparent discriminative ability. Notably, while the baseline surgical risk was 4%, patients with a TALAR score ≥ 2 demonstrated a 17.5% conversion rate to surgery. This represents a more than four-fold increase in risk compared to the general cohort.

Eversion mobility emerged as the only range-of-motion parameter associated with later surgical indication. Increased eversion of the affected ankle may reflect a potential concomitant lesion of the medial compartment and the deltoid ligament, contributing to greater mechanical joint instability [19]. This hypothesis is supported by biomechanical studies demonstrating that deltoid ligament injury significantly destabilizes the ankle joint, with complete deltoid tear causing severe instability [20]. Furthermore, Roemer et al. found that concomitant medial compartment damage is common in severe lateral ankle sprains [21].

Similarly, reduced plantar flexor strength and pain during dorsiflexion were significantly associated with subsequent surgical treatment. The exact mechanisms underlying these clinical signs remain speculative, as our study lacks imaging data to confirm specific structural lesions. While these symptoms at two weeks post-injury could simply reflect acute residual edema or post-traumatic guarding, they might also represent early signs of posterior inflammation [22,23] or anterior translation of the talus resulting from the ligamentous injury [24,25]. This translation typically triggers pain during dorsal flexion, while the observed loss of plantar flexion strength may be secondary to the acute pain elicited by tension on the newly ruptured ligament.

More importantly, we hypothesize that these early clinical ‘red flags’ might actually unmask a pre-existing, unrecognized Chronic Ankle Instability (CAI). A significant proportion of patients may harbor compromised ankle function without clearly recalling previous minor sprains. In such cases, the acute event exacerbates a subclinical instability. Therefore, the parameters included in the TALAR score might not solely measure the severity of the acute tissue damage, but effectively identify this ‘acute-on-chronic’ patient phenotype. This would explain their rapid failure of conservative management and the ultimate need for surgical stabilization. When combined into the TALAR score, these three features provided a substantially superior discriminative capacity for identifying patients who progressed to surgical treatment than any single variable alone.

The high AUC obtained for the composite score suggests that a multidimensional approach integrating mobility, strength, and pain is more informative than isolated measurements. This finding aligns with the SPRAINED prognostic model, developed by Keene et al., which demonstrated that combining multiple clinical variables (age, BMI, pain at rest, pain bearing weight, ability to bear weight) can predict poor functional recovery at 9 months defining as persistent pain, functional difficulty, or lack of confidence in the ankle, rather than the need for surgical intervention [26,27].

It is critical to emphasize that our primary endpoint—subsequent surgical management—is a clinical decision rather than a purely objective biological outcome. Although surgical indications followed a strict institutional protocol based on failed conservative care, they can be heavily influenced by patient-specific factors, such as athletic expectations, varying levels of adherence to the rehabilitation protocol, and lifestyle demands. Furthermore, because this is an exploratory study, a formal Decision Curve Analysis (DCA) was not conducted. This limits our ability to evaluate the clinical net benefit and the specific impact of false positives. Future larger-scale studies should establish the Number Needed to Screen (NNS) to fully determine the cost-effectiveness and practical utility of the TALAR score in routine triage.

The TALAR score represents a fundamentally different and novel approach in literature. While the SPRAINED model focuses on subjective symptoms and general recovery parameters, the TALAR score integrates objective functional assessments, such as eversion mobility, plantar flexor strength, and pain during dorsiflexion, that reflect specific biomechanical and structural deficits.

In the present cohort, the TALAR score showed promising apparent discrimination, with an AUC of 0.889. After bootstrap correction for optimism, the AUC remained promising at 0.855, suggesting that the observed performance was not entirely explained by overfitting. Furthermore, the observed surgical conversion rate increased progressively across score levels, from 0% in patients with a score of 0, to 1.3% for score 1, 13.9% for score 2, and 40.0% for score 3. This risk gradient supports the potential value of the score as a simple tool for identifying patients who may require closer follow-up after acute ankle sprain.

However, these findings should not be interpreted as validation of a clinical decision tool for indicating surgery. The endpoint of surgical stabilization remains a clinical decision influenced by symptoms, functional demands, rehabilitation response, and patient expectations. Therefore, the TALAR score should currently be considered a hypothesis-generating risk-stratification framework rather than a validated prognostic model. Its potential role would be to support early recognition of patients at higher risk of conservative treatment failure, not to replace clinical judgment or define surgical indication.

Future research should prioritize multicenter prospective validation across larger and more diverse cohorts to verify the reliability of these predictors. Furthermore, it remains essential to assess the calibration performance of the score and its tangible clinical utility when routinely implemented to guide clinical management strategies.

## 5. Limitations

Several limitations must be acknowledged. The cut-off values were derived from the same cohort and therefore they require external validation before clinical implementation. Information regarding rehabilitation adherence and activity level during follow-up was not available, which may have influenced outcomes. Additionally, only a small number of patients ultimately underwent surgical treatment, which may limit the statistical power and generalizability of comparisons between surgical and non-surgical groups.

Furthermore, the nature of our primary endpoint must be considered. Although the indication for surgery was strictly standardized in our center (requiring at least 3 months of failed conservative management and persistent symptoms), ‘subsequent surgical management’ is intrinsically a clinical decision rather than a purely objective biomechanical measure. As such, the endpoint may still be partially influenced by patient-specific factors—such as athletic expectations, varying levels of adherence to the rehabilitation protocol, and lifestyle demands—which could introduce a degree of bias. Furthermore, a significant limitation is that the initial grade of the ankle sprain at the time of injury was not considered in the analysis. This lack of baseline severity stratification may overlook a critical prognostic factor. Integration of this score into follow-up protocols after acute ankle sprain could allow for risk-adapted management strategies, closer monitoring of high-risk patients, and earlier referral when persistent instability is suspected.

## 6. Conclusions

Early functional and pain-related impairments after acute ankle sprain are associated with the subsequent development of chronic instability requiring surgical treatment. Among all evaluated variables, increased eversion mobility, reduced plantar flexor strength, and pain during dorsal flexion were the only significant univariable clinical predictors of poor outcome. Combined within the TALAR score, these parameters demonstrated a strong discriminative capacity for identifying patients who ultimately required surgery. The TALAR score represents a simple, clinically applicable tool that may help identify patients at higher risk of progression to chronic ankle instability and supports the need for prospective validation in larger populations.

## Figures and Tables

**Table 1:** Demographic characteristics.

Characteristic	Overall Population	Non-Surgical Population	Surgical Population	*p* Value
Mean Age (±SD)	36.12 (±15.84)	36.12 (±15.85)	36.00 (±15.22)	0.1
Number of patients	197	189	8	-
Sex (M/F)	91/106	88/101	3/5	0.7
BMI (±SD)	23.75 (±7.8)	23.80 (±7.9)	22.66 (±3.2)	0.4
Affected side (DX/SX)	114/83	109/80	5/3	1.0

**Table 2:** Test results in the Non-surgical group and Surgical group. (a = affected ankle, h = Healthy ankle; Nr: number of repetitions; mV: millivolt; N: Newton).

Variable	Non Surg–Median [IQR]	Surg–Median [IQR]	*p* (Mann–Whitney)
Eversion (°) (a)	10 [10–15]	20 [13.8–24]	0.009
Plantar flexors (Kg) (a)	20 [14–30]	13 [10.8–15.5]	0.026
Dorsiflexion deficit (°)	−5 [−10–0]	−10 [−16.2–−7.25]	0.07
Dorsal flexors (Kg) (a)	15 [11–21]	12.5 [8–14.2]	0.09
Plantar flexors (Kg) (h)	23.5 [17–32.8]	17 [15–21.2]	0.11
Eversion (°) (h)	15 [10–20]	20 [17.5–28.8]	0.12
Dorsiflexion (°) (h)	12 [5–20]	20 [16–21.2]	0.14
Inversion (°) (h)	30 [20–35]	32.5 [27.2–40]	0.15
6 m Timed Hop (Nr) (a)	3 [2.42–3.75]	3.5 [3.5–3.76]	0.15
6 m Timed Hop (Nr) (h)	3 [2.29–4]	3.5 [3.45–4.15]	0.2
Foot-lift Test (N) (a)	10 [6–15]	7 [4.5–9.5]	0.22
Dorsal flexors (Kg) (h)	17 [12–26.8]	14 [11–17.2]	0.22
Foot-lift Test (N) (h)	8 [5–12]	4 [4–8]	0.23
Faam (Daily activities)	0.821 [0.631–0.929]	0.672 [0.622–0.75]	0.25
Inversion (°) (a)	20 [20–30]	25 [23.8–30]	0.27
Plantar flexion (°) (h)	65 [56–70]	75 [57.5–82.5]	0.28
Peroneal muscles (Kg) (a)	10 [6–16]	8.5 [7–9]	0.28
Posterolateral deficit (cm)	−2.41 [−7.12–1.56]	−0.25 [−2.56–1.88]	0.29
Plantar Deficit (kg) (%)	−12.5 [−29.4–0]	−22.2 [−35.5–−11.2]	0.33
Posteromedial Deficit (cm)	−2 [−6.87–0.812]	−5.5 [−6.75–−3.37]	0.34
Plantar flexion (°) (a)	60 [50–70]	70 [45–81.2]	0.35
Inversion Deficit (°)	0 [−10–0]	−6.5 [−11.2–0]	0.35
Anterior (cm) (a)	50.9 [44–55.9]	53 [49.4–57.5]	0.37
Gastrocnemius deficit (cm)	−0.5 [−1–0]	0 [−0.25–0]	0.41

**Table 3:** VAS analysis (Fisher’s exact test).

VAS	N. Non Surg.	N Surg.	*p* Value
Dorsiflexion	32/189 (16.9%)	5/8 (62.5%)	0.007
Plantarflexion	39/189 (20.6%)	3/8 (37.5%)	0.37
Inversion	39/189 (20.6%)	3/8 (37.5%)	0.37
Eversion	30/189 (15.9%)	3/8 (37.5%)	0.134

**Table 4:** Diagnostic performance of the TALAR score.

Score	N° Non-Surgical	N° Surgical	Sensitivity	Specificity	PPV
SCORE 0	79	0	NA	NA	NA
SCORE 1	77	1	1.00	0.405	0.068
SCORE 2	30	5	0.875	0.822	0.175
SCORE 3	3	2	0.250	0.984	0.400

## Data Availability

All the data we analyzed and tables we compiled are available for any clarification.
